# Supplementary material for: The Evolutionary History of Vertebrate Adhesion GPCRs and Its Implication on Their Classification
Source: Int J Mol Sci. 2021 Oct 30;22(21):11803. doi: 10.3390/ijms222111803 (PMC8584163; doi:10.3390/ijms222111803)
Supplement: Supplementary file 1 [file ijms-22-11803-s001.zip › Wittlake et al_final Suppl information.pdf]

The Evolutionary History of Vertebrate Adhesion GPCRs and Its Implication on Their Classification

Aline Wittlake [1], Simone Prömel [1,2,*], and Torsten Schöneberg [1,*]

[1] Division of Molecular Biochemistry, Rudolf Schönheimer Institute of Biochemistry, Medical Faculty, Leipzig University, 04103 Leipzig, Germany; a.j.wittlake@gmx.de
[2] Department of Biology, Institute of Cell Biology, Heinrich Heine University Düsseldorf, 40225 Düsseldorf, Germany
* Correspondence: proemel@uni-duesseldorf.de (S.P.); schoberg@medizin.uni-leipzig.de (T.S.); Tel.: +49-341-972-2150 (T.S.)

Supplementary Information

class	species	abbr.	pictogram	order	Annotation release
Mammilia	Homo sapiens	hs		primates	109
	Mus musculus	mm		rodentia	108
	Monodelphis domestica	md		Didelphimorphia	103
	Mytois lucifugus	ml		Chiroptera	102
	Ornithorhynchus anatinus	oa		Monotremata	104
	Pteropus alecto	pa		Chiroptera	102
Aves	Anas platyrhynchos	ap		Anseriformes	103
	Aptenodytes forsteri	af		Sphenisciformes	101
	Apteryx mantelli	am		Apterygiformes	100
	Calypte anna	ca		Apodiformes	101
	Charadrius vociferus	cv		Charadriiformes	100
	Cuculus canorus	cc		Cuculiformes	100
	Columba livia	cl		Columbiformes	102
	Dromaius novaehollandiae	dn		Casuariiformes	100
	Falco peregrinus	fp		Falconiformes	102
	Gallus gallus	gg		Galliformes	104
	Taeniopygia guttata	tg		Passeriformes	105
	Tyto alba	ta		Strigiformes	101
Sauria (including Archelosauria and Lepidosauria)	Alligator mississippiensis	alm		Crocodylia	102
	Anolis carolinensis	ac		Squamata	102
	Python bivittatus	pb		Squamata	102
	Terrapene carolina triunguis	tct		Testudines	102
Amphibia	Nanorana parkeri	np		Anura	100
	Rhinatrema bivittatum	rb		Gymnophiona	100
	Xenopus tropicalis	xp		Anura	104
Actinopteri (bony fish)	Danio rerio	dr		Cypriniformes	106
	Takifugu rubripes	tr		Tetraodontiformes	103
Transition species					
Leptocardii	Branchiostoma belcheri	bb		Amphioxiformes	100
Ascidiacea (Tunicata)	Ciona intestinalis	ci		Enterogona	104
Hyperoartia	Petromyzon marinus	pm		Petromyzontiformes	100

table 1 - all analyzed species

Legend of the animal illustrations used in suppl. Figure S1

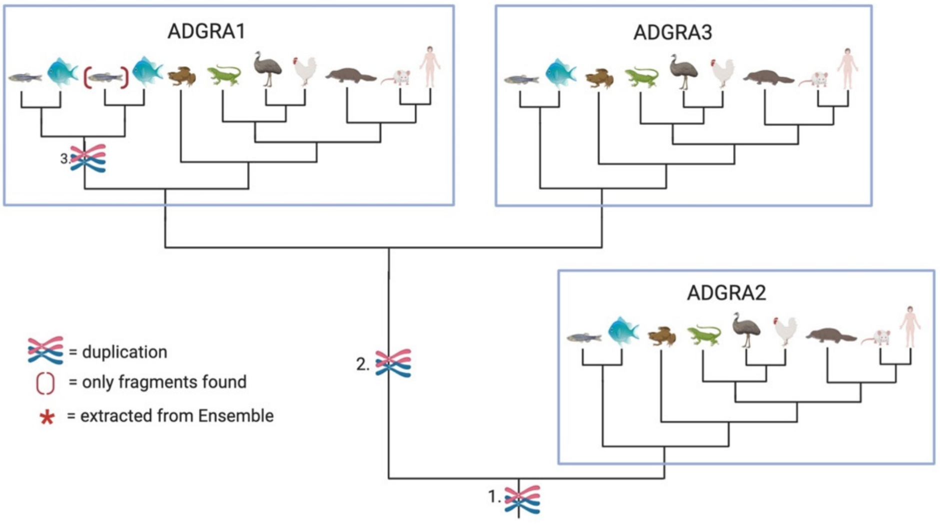

suppl. Figure S1a

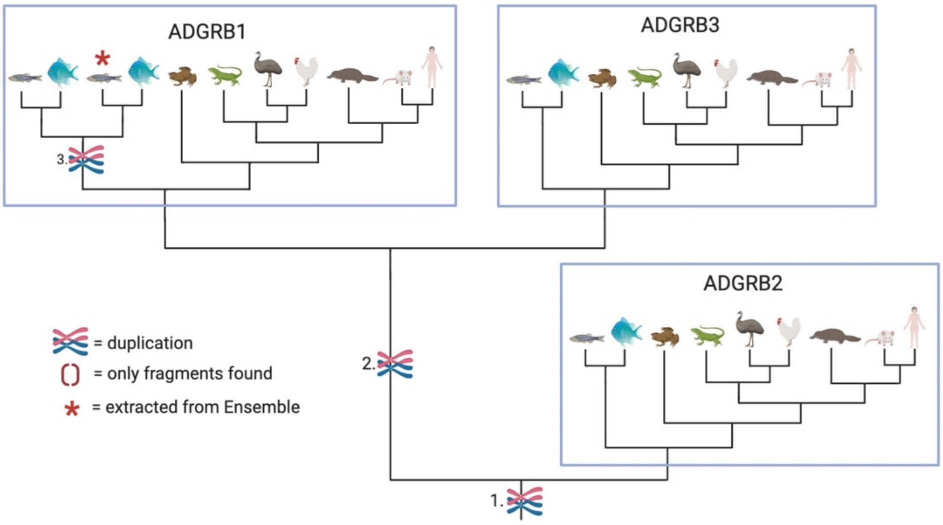

suppl. Figure S1b

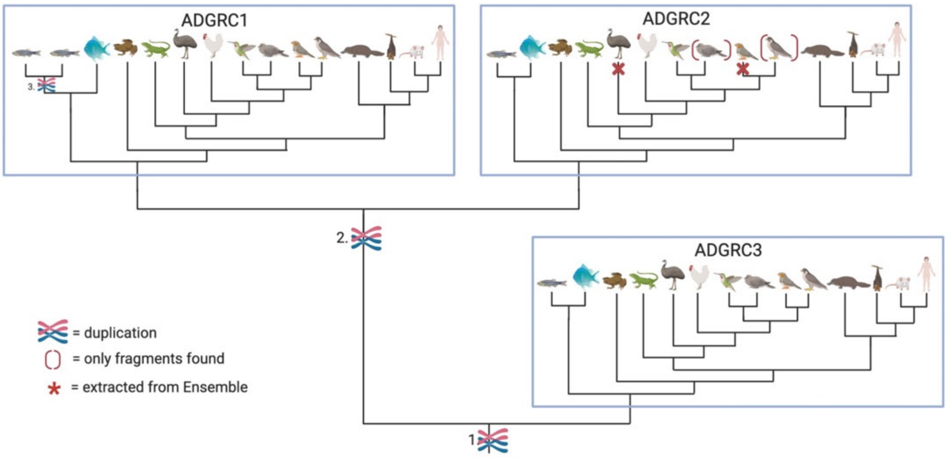

species	abbr.	pictogram	order	neo-/palaeo-gnathae	found in this study	sequence corrected by NCBI to prevent frameshifts
Anas platyrhynchos	ap		Anseriformes	Neo	exodomain	-
Aptenodytes forsteri	af		Sphenisciformes	Neo	exodomain	-
Apteryx mantelli	am		Apterygiformes	Palaeo	exodomain	-
Calypte anna	ca		Apodiformes	Neo	GPS+7tm	2 bases added and 1 deleted
Charadrius vociferus	cv		Charadriiformes	Neo	exodomain	-
Cuculus canorus	cc		Cuculiformes	Neo	GPS+ ?tms	-
Columba livia	cl		Columbiformes	Neo	exodomain	-
Dromaius novaehollandiae	dn		Casuariiformes	Palaeo	exodomain	-
Falco peregrinus	fp		Falconiformes	Neo	GPS+ ?tms	2 bases added and 2 deleted
Gallus gallus	gg		Galliformes	Neo	GPS+7tm	-
Taeniopygia guttata	tg		Passeriformes	Neo	exodomain	-
Tyto alba	ta		Strigiformes	Neo	exodomain	-

table 3- ADGRC2 in Aves

suppl. Figure S1c

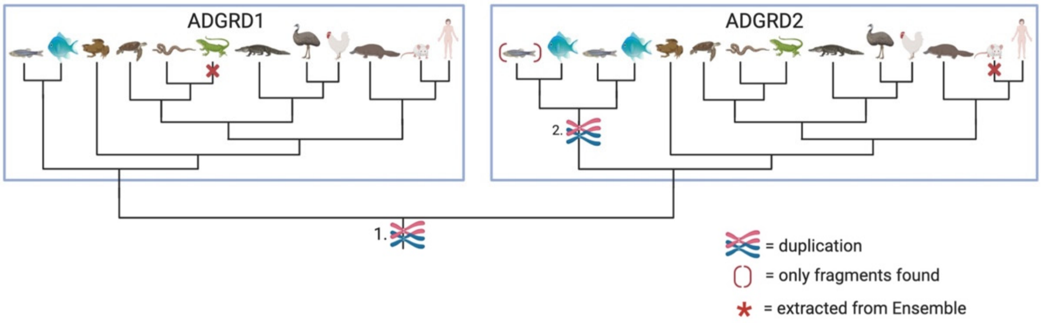

suppl. Figure S1d

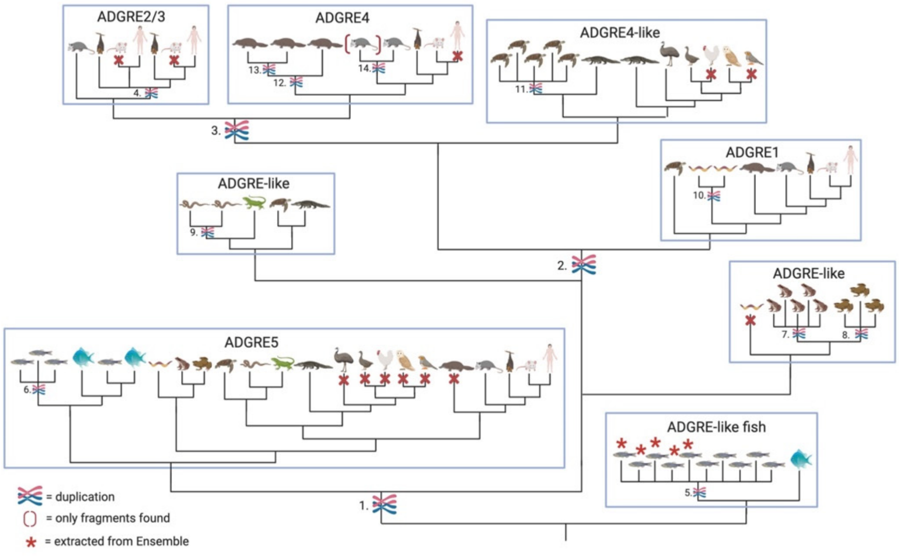

species	abbr.	pictogram	order	neo-/palaeo-gnathae	found in this study
Anas platyrhynchos	ap		Anseriformes	Neo	GPS+7tm
Aptenodytes forsteri	af		Sphenisciformes	Neo	-
Apteryx mantelli	am		Apterygiformes	Palaeo	GPS+7tm
Calypte anna	ca		Apodiformes	Neo	-
Charadrius vociferus	cv		Charadriiformes	Neo	GPS+7tm
Cuculus canorus	cc		Cuculiformes	Neo	GPS+7tm
Columba livia	cl		Columbiformes	Neo	-
Dromaius novaehollandiae	dn		Casuariiformes	Palaeo	GPS+7tm
Falco peregrinus	fp		Falconiformes	Neo	GPS+7tm
Gallus gallus	gg		Galliformes	Neo	-
Taeniopygia guttata	tg		Passeriformes	Neo	-
Tyto alba	ta		Strigiformes	Neo	GPS+7tm

table 4- ADGRE-like in Aves

suppl. Figure S1e

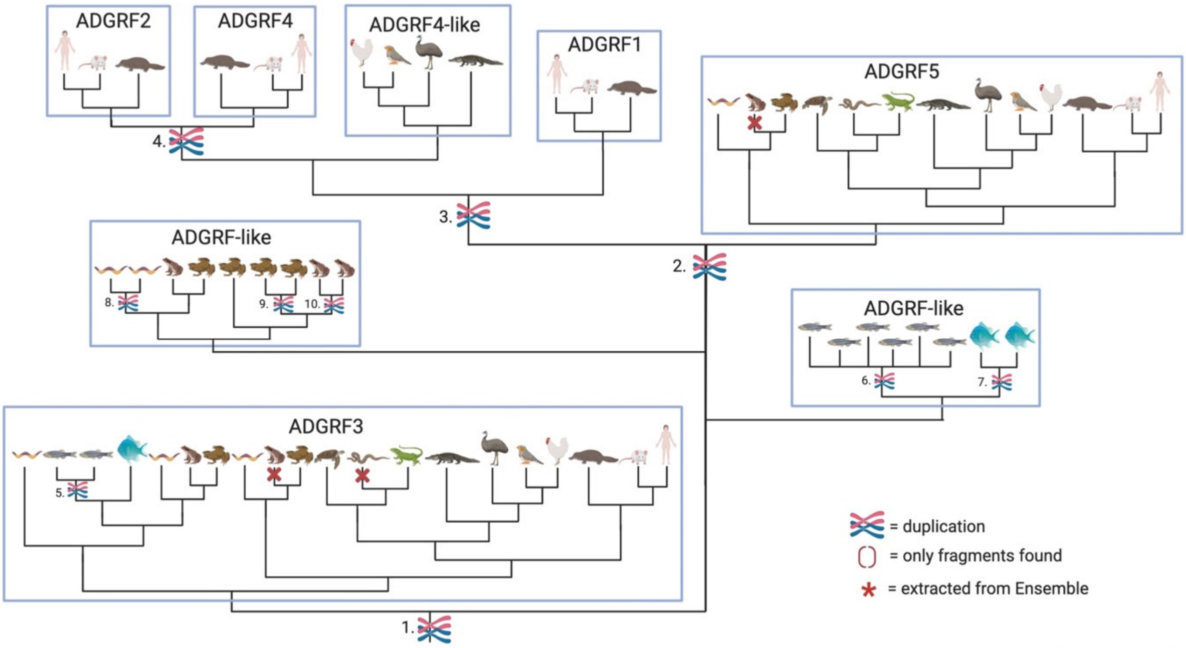

suppl. Figure S1f

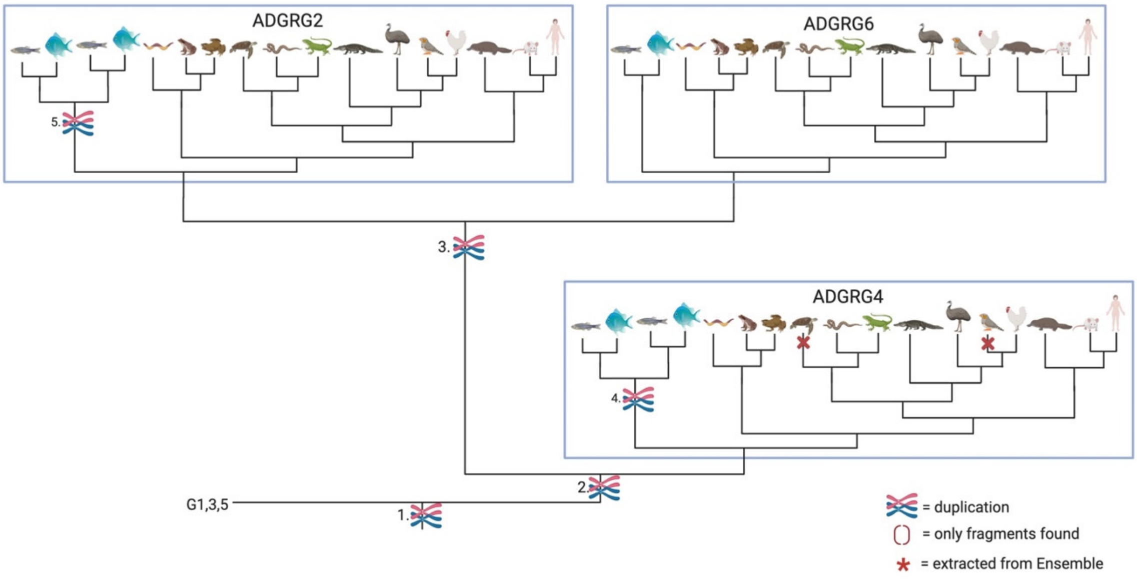

suppl. Figure S1g

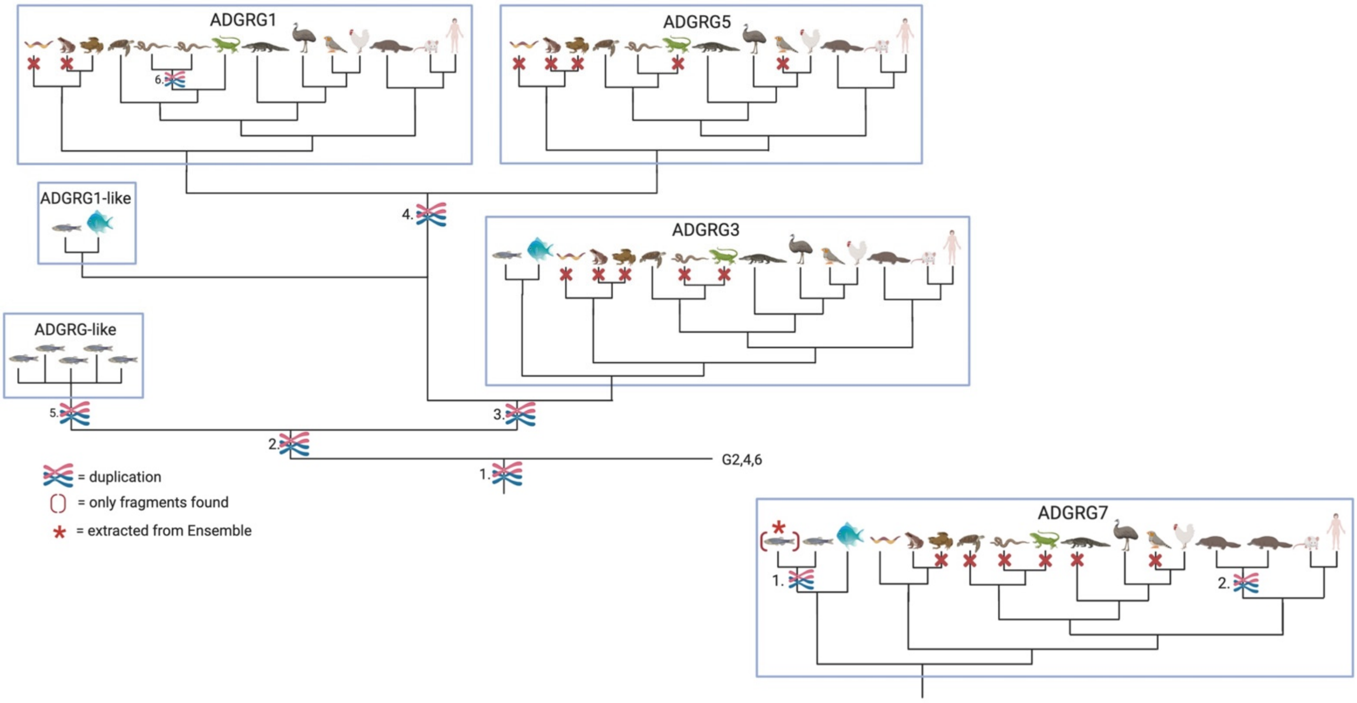

suppl. Figure S1g

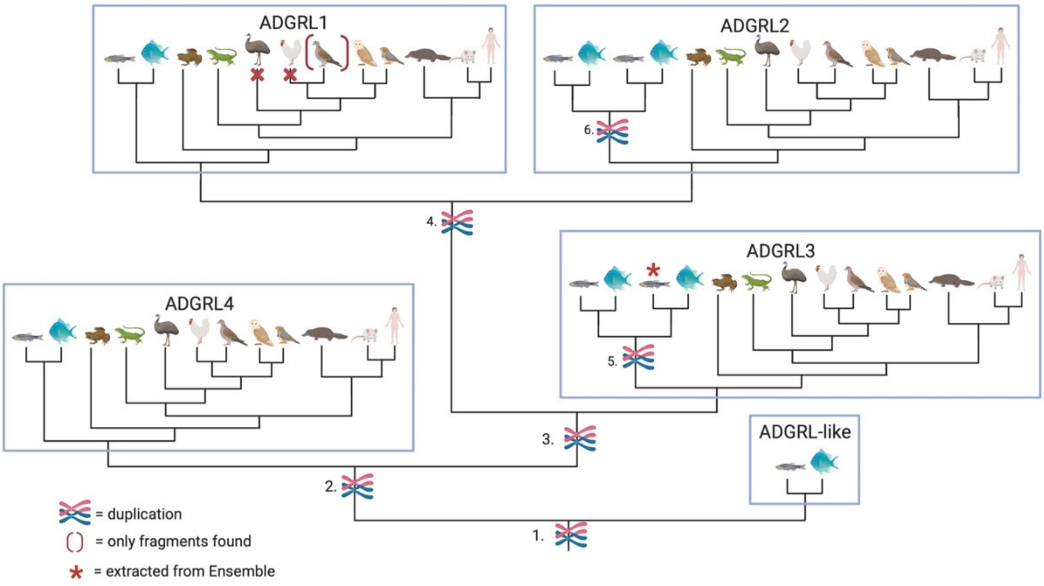

suppl. Figure S1h

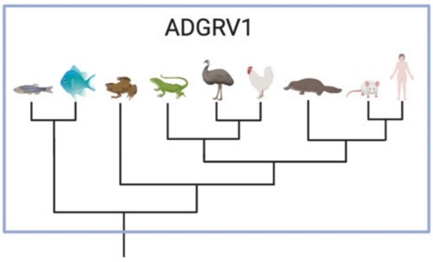

suppl. Figure S1i

Suppl. Figure S1. Schematic presentation of the phylogeny of all aGPCR families. Figure S1. generated with BioRender.

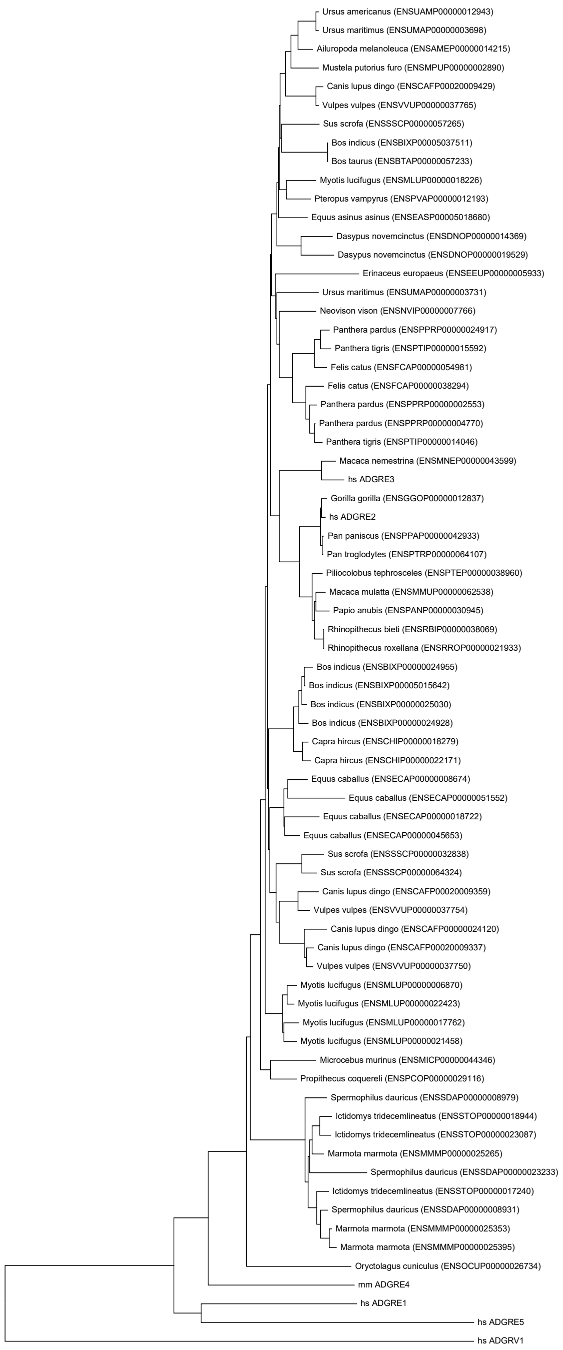

Suppl. Figure S2. Phylogeny of mammalian EMR2/ADGRE2. The phylogenetic relation of selected mammalian ADGRE2 orthologs and human EMR1/ADGRE1, EMR3/ADGRE3, EMR4/ADGRE4, CD97/ADGRE5, and VLGR1/ADGRV1 are given. There are several duplications of EMR2/ADGRE2 orthologs in carnivores, marmots, horses, and bats. human (hs, *Homo sapiens*), mouse (mm, *Mus musculus*).

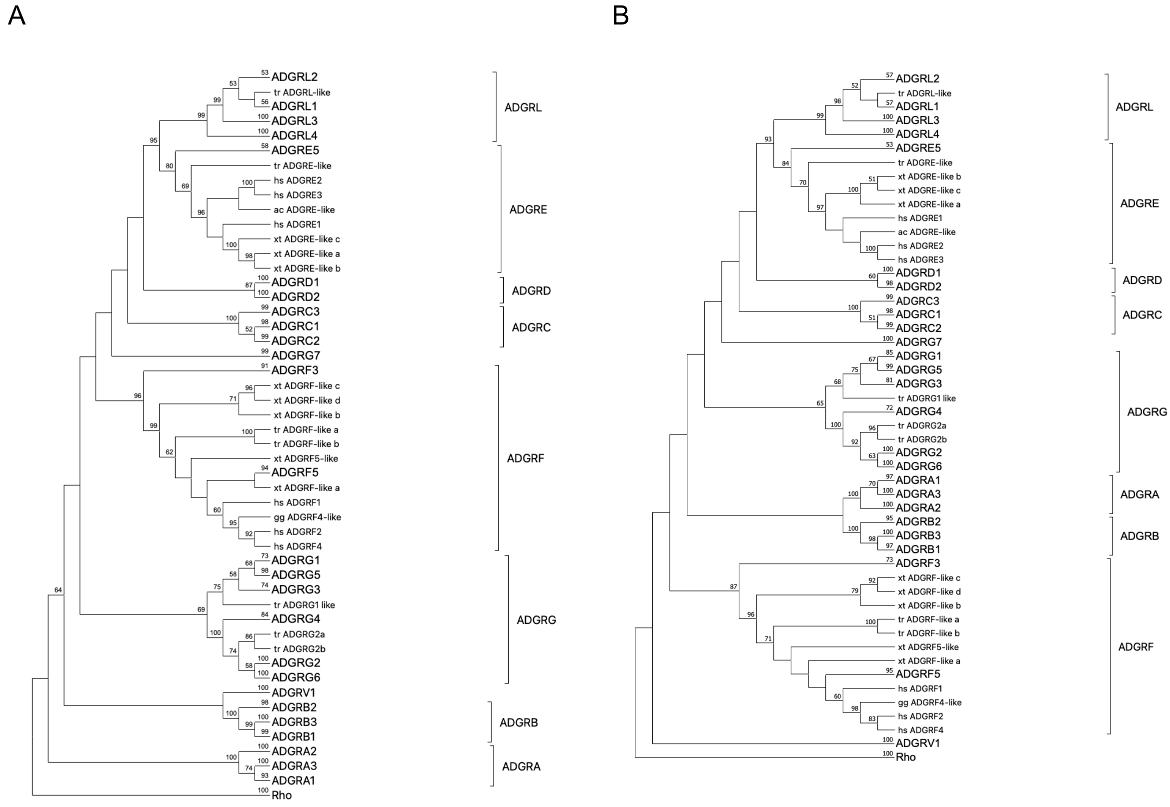

Suppl. Figure S3. *Phylogeny of representative vertebrate aGPCRs.* The 7TM domain amino acid sequence of representative vertebrate aGPCRs were aligned with MUSCLE (A) and ClustalW (B). The evolutionary history was inferred using the Neighbor-Joining method [1] based on a sequence alignment of the 7TM part of vertebrate aGPCR orthologs. Rhodopsin orthologs (Rho) served as outgroup. The optimal tree is shown. The percentage of replicate trees in which the associated taxa clustered together in the bootstrap test (1,000 replicates) are shown next to the branches [2]. The evolutionary distances were computed using the Poisson correction method [3] and are in the units of the number of amino acid substitutions per site. This analysis involved 148 amino acid sequences. All ambiguous positions were removed for each sequence pair (pairwise deletion option). Evolutionary analyses were conducted in MEGA [4,5]. The subtrees of currently and newly assigned aGPCR families were condensed and labeled with a larger font size. human (hs, *Homo sapiens*), chicken (gg, *Gallus gallus*), lizard (ac, *Anolis carolinensis*), frog (xt, *Xenopus tropicalis*), and bony fish (tr, *Takifugu rubripes*).

A

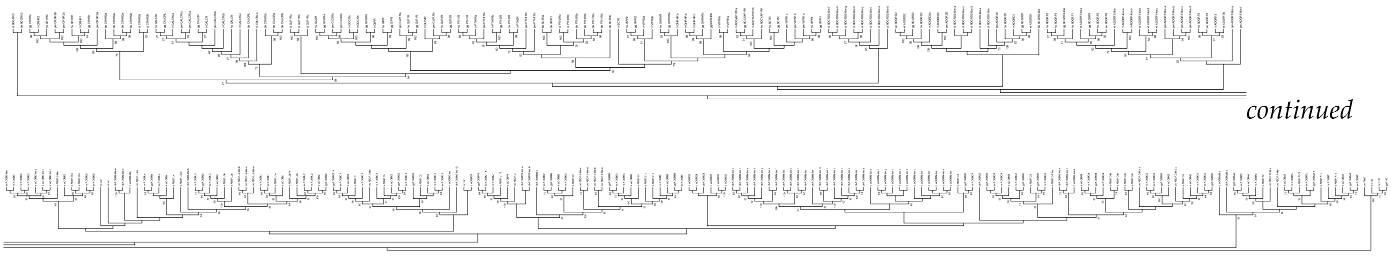

B

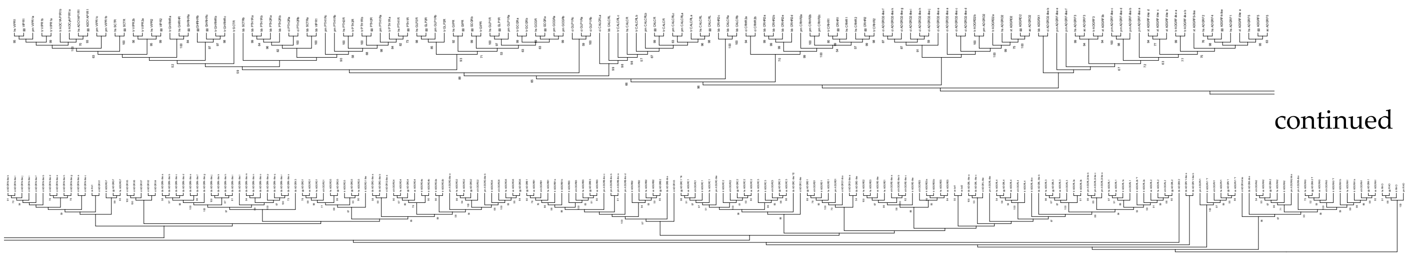

Suppl. Figure S4. *Phylogenetic tree of representative Chordata and C. elegans aGPCRs and secretin-like GPCRs.* The 7TM domain amino acid sequence of representative *Chordata* and *C. elegans* aGPCRs and secretin-like GPCRs were aligned with MUSCLE (A) and ClustalW (B). The evolutionary history was inferred using the Neighbor-Joining method [1] based on a sequence alignment of the 7TM part of chordate and *C. elegans* aGPCR orthologs. Rhodopsin orthologs (Rho) served as outgroup. The optimal tree is shown. The percentage of replicate trees, in which the associated taxa clustered together in the bootstrap test (1,000 replicates) are shown next to the branches [2]. The evolutionary distances were computed using the Poisson correction method [3] and are in the units of the number of amino acid substitutions per site. This analysis involved 338 amino acid sequences. All ambiguous positions were removed for each sequence pair (pairwise deletion option). Evolutionary analyses were conducted in MEGA [4,5]. The subtrees of currently and newly assigned aGPCR families and the secretin-like receptors were condensed and labeled with a larger font size. *C. elegans*, as a distantly related invertebrate with the well-studied aGPCR members latrophilin 1 and 2 (lat-1, lat-2) and flamingo (fmi) [6], was included to internally evaluated the rooting of the trees. Thus, latrophilins were expected to cluster with vertebrate LPHN/AGDRL (see Figure 1) and flamingo was currently not well-assigned to a vertebrate aGPCR family. Secretin-like receptors are clear descents of ADGRD2 as supported by both trees. Condenced trees are given in Figure 5. lamprey (pm, *Petromyzon marinus*), lancelet (bb *Branchiostoma belcheri*), vase tunicate (ci, *Ciona intestinalis*), nematode (ce, *Caenorhabditis elegans*).

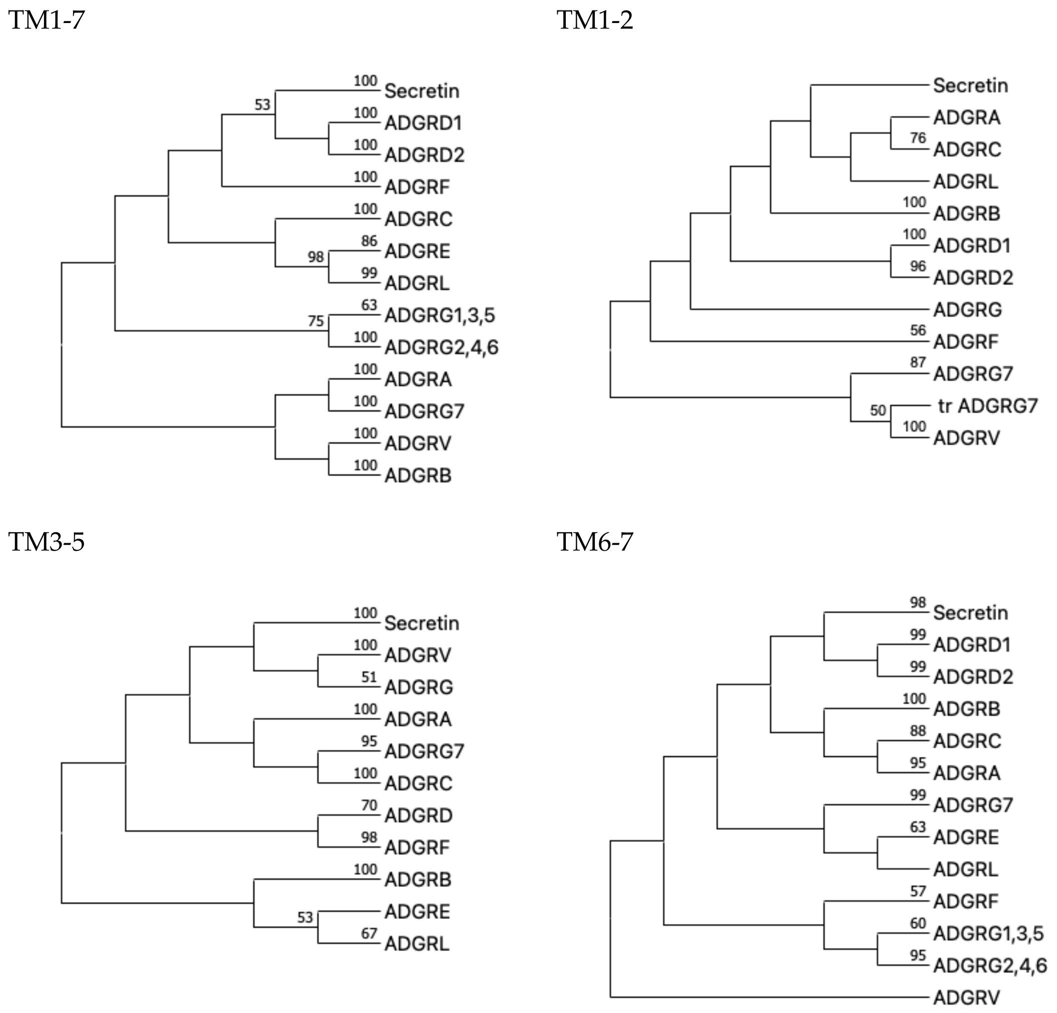

Suppl. Figure S5. *Phylogenetic relation of representative vertebrate aGPCRs and secretin-like 7TM domains and partial TM receptor sequences.* The amino acid sequence of representative vertebrate aGPCRs (human (hs, *Homo sapiens*), chicken (gg, *Gallus gallus*), lizard (ac, *Anolis carolinensis*), frog (xt, *Xenopus tropicalis*), pufferfish (tr, *Takifugu rubripes*) were aligned with MUSCLE. The evolutionary history was inferred using the Neighbor-Joining method [1]. The bootstrap consensus tree inferred from 1000 replicates [2] is taken to represent the evolutionary history of the taxa analyzed. Branches corresponding to partitions reproduced in less than 50% bootstrap replicates are collapsed. The percentage of replicate trees in which the associated taxa clustered together in the bootstrap test (1,000 replicates) are shown above the branches [2]. The evolutionary distances were computed using the Poisson correction method [3] and are in the units of the number of amino acid substitutions per site. This analysis involved 201 amino acid sequences. All ambiguous positions were removed for each sequence pair (pairwise deletion option). Evolutionary analyses were conducted in MEGA [4,5].

Suppl. Table S1. ***Sequences retrieved from Ensemble instead of GenBank*** (excel file).

Table S2. ***Mutational constraint in human aGPCRs.*** Data were taken from gnomAD v2.1.1 and are based on 125,748 exomes and 15,708 genomes from unrelated individuals sequenced as part of various disease-specific and population genetic studies aligned against the human GRCh37 reference [7]. The average loss-of function (LoF) o/e ratio of all aGPCRs is 0.5. The average o/e ratio of ADGRA, B, C, and L (blue fields, LoF e/o ratio = 0.21, p = 0.001) is significantly below and the average o/e ratio of ADGRE, and F (orange fields, LoF o/e ratio = 0.85, p = 0.02) is significantly above the o/e ratio of all aGPCRs (o/e ratio = 0.5). Of note, the constraint data are derived from human genomic sequences and, therefore, represent only the constraint in humans in its ecological environment.

aGPCR	o/e score synonymous	o/e score missense	o/e score pLoF
ADGRA1	0.93	0.8	0.36
ADGRA2	0.99	0.8	0.42
ADGRA3	1.05	0.95	0.23
ADGRB1	1.05	0.58	0.06
ADGRB2	0.92	0.59	0.15
ADGRB3	1.08	0.82	0.07
ADGRC1	1.15	0.92	0.17
ADGRC2	1.08	0.86	0.17
ADGRC3	0.91	0.95	0.17
ADGRD1	1.05	0.96	0.6
ADGRD2	0.89	0.88	0.69
ADGRE1	0.98	0.93	0.86
ADGRE2	0.96	0.92	0.97
ADGRE3	0.89	0.89	1.13
ADGRE5	1.04	0.9	0.4
ADGRF1	1.16	1.04	0.93
ADGRF2	1.13	1.09	1.38
ADGRF3	1.02	0.93	0.87
ADGRF4	1.13	1.08	0.86
ADGRF5	1.04	0.93	0.26
ADGRG1	1.11	1.01	0.61
ADGRG2	0.96	0.72	0.17
ADGRG3	0.96	0.95	0.87
ADGRG4	1.1	1.07	0.92
ADGRG5	0.98	0.94	0.26
ADGRG6	0.95	0.87	0.32
ADGRG7	1.08	0.95	0.83
ADGRL1	1.11	0.86	0.1
ADGRL2	1.04	0.87	0.04

ADGRL3	1.01	0.73	0.09
ADGRL4	1.12	1	0.7
ADGRV1	1.02	1	0.44

References

1. Saitou, N.; Nei, M. The neighbor-joining method: a new method for reconstructing phylogenetic trees. *Mol. Biol. Evol.* **1987**, *4*, 406–425, doi:10.1093/oxfordjournals.molbev.a040454.
2. Felsenstein, J. Confidence Limits on Phylogenies: An Approach Using the Bootstrap. *Evolution* **1985**, *39*, 783–791, doi:10.1111/j.1558-5646.1985.tb00420.x.
3. Zuckerkandl, E.; Pauling, L. *Evolutionary Divergence and Convergence in Proteins*; Academic Press: Cambridge, MA, USA, 1965; pp. 97–166.
4. Tamura, K.; Stecher, G.; Kumar, S. MEGA11: Molecular Evolutionary Genetics Analysis version 11. *Mol. Biol. Evol.* **2021**, *38*, 3022–3027, doi:10.1093/molbev/msab120.
5. Stecher, G.; Tamura, K.; Kumar, S. Molecular Evolutionary Genetics Analysis (MEGA) for macOS. *Mol. Biol. Evol.* **2020**, *37*, 1237–1239, doi:10.1093/molbev/msz312.
6. Langenhan, T.; Prömel, S.; Mestek, L.; Esmaeili, B.; Waller-Evans, H.; Hennig, C.; Kohara, Y.; Avery, L.; Vakonakis, I.; Schnabel, R.; et al. Latrophilin signaling links anterior-posterior tissue polarity and oriented cell divisions in the C. elegans embryo. *Dev. Cell* **2009**, *17*, 494–504, doi:10.1016/j.devcel.2009.08.008.
7. Karczewski, K.J.; Francioli, L.C.; Tiao, G.; Cummings, B.B.; Alföldi, J.; Wang, Q.; Collins, R.L.; Laricchia, K.M.; Ganna, A.; Birnbaum, D.P. et al. Author Correction: The mutational constraint spectrum quantified from variation in 141,456 humans. *Nature* **2021**, *590*, E53, doi:10.1038/s41586-020-03174-8.
